# Optically Probing the Chirality of Single Plasmonic Nanostructures and of Single Molecules: Potential and Obstacles

**DOI:** 10.1021/acsphotonics.2c01205

**Published:** 2022-10-20

**Authors:** Subhasis Adhikari, Michel Orrit

**Affiliations:** Huygens-Kamerlingh Onnes Laboratory, Leiden University, Niels Bohrweg 2, 2333 CALeiden, Netherlands

**Keywords:** chirality, optical rotatory dispersion, plasmon-coupled CD, photothermal circular dichroism, single nanoparticles, magnetic CD

## Abstract

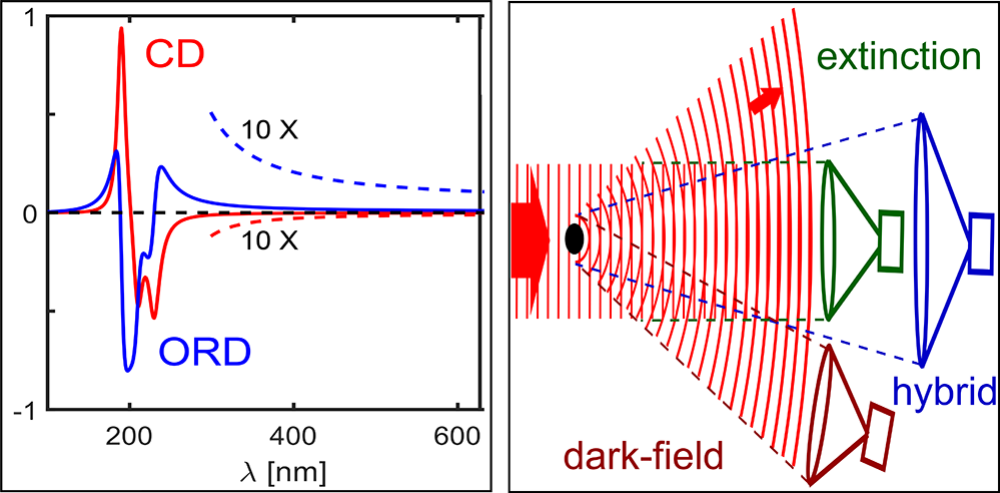

Circular dichroism (CD) is a standard method for the analysis of biomolecular conformation. It is very reliable when applied to molecules, but requires relatively large amounts of solution. Plasmonics offer the perspective of enhancement of CD signals, which would extend CD spectrometry to smaller amounts of molecules and to weaker chiral signals. However, plasmonic enhancement comes at the cost of additional complications: averaging over all orientations is no longer possible or reliable, linear dichroism leaks into CD signals because of experimental imperfections, scattering and its interference with the incident beam must be taken into account, and the interaction between chiral molecules and possibly chiral plasmonic structures considerably complicates the interpretation of measured signals. This Perspective aims to explore the motivations and problems of plasmonic chirality and to re-evaluate present and future solutions.

## Introduction

Chiral molecules are molecules whose mirror image cannot be superimposed with the original.^1^ The two forms, which are mirror images of each other, are called enantiomers. Many organic chiral molecules possess at least one asymmetric carbon, that is, a sp^3^ carbon with four different substituents. Although they have closely related chemical structures and properties, the chemical properties of enantiomers often differ radically when they interact with other chiral molecules, in particular, in biochemical reactions. Most complex biomolecules are chiral, for example, amino acids are of the l-enantiomeric type, whereas sugars are of the d-enantiomeric type.^2^ Enantioselective detection of chiral molecules is therefore a holy grail for biochemical and pharmaceutical application, as only one enantiomer of a drug is active, while the other enantiomer is often inactive or downright harmful.^3^

The optical effects of chirality in organic molecules are generally weak, not only because molecules are small compared to the light wavelength, but because of the low electronic velocities (*v*) compared to light velocity (*c*). The circular dichroism (CD) cross section of a transition scales as a scalar product of its electronic and magnetic dipole moments:^4,5^
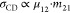
1

Optical effects of chirality are thus reduced by a factor ∼ *v*/*c* when compared to electronic absorption, and this factor is very small for molecules consisting of light atoms. Therefore, most optical enantioselective methods require measurements of large ensembles of chiral molecules, either of their circular dichroism (CD) or of their optical rotatory dispersion (ORD, also called circular birefringence, CB). Circular dichroism is the differential absorption of left- and right-circularly polarized light by chiral molecules, whereas ORD gives rise to a rotation of the polarization direction when linearly polarized light propagates through a chiral medium. CD and ORD are respectively the imaginary and real part of a matrix element of the dielectric permittivity tensor. Therefore, the CD spectrum of molar ellipticity θ(λ) and the ORD spectrum of molar rotation φ(λ), expressed in the same units, are related by Kramers–Kronig relations of the following type:^6^
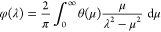
2

**Figure 1:**
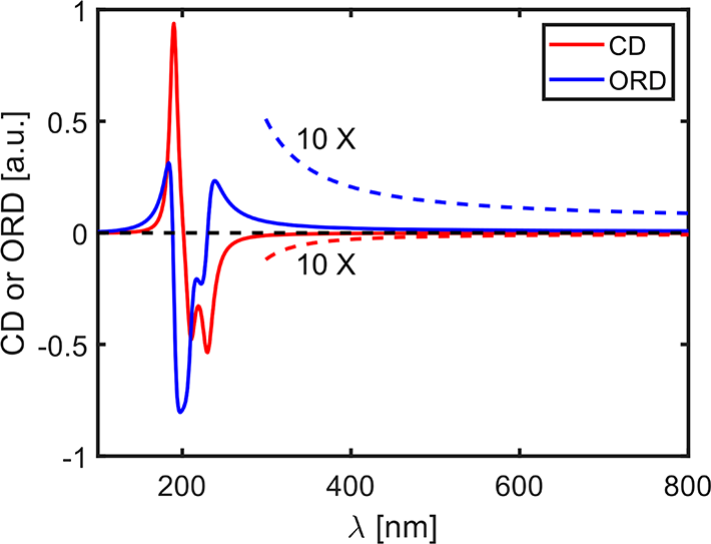
CD vs ORD spectra. The red solid line is a model of the CD spectrum of an α helix and the blue solid line is its ORD spectrum calculated using the equations for CD and ORD given in ref (7). CD and ORD are related by the Kramers–Kronig relation. The dashed spectra, magnified 10 times, show that the ORD value is significant in the visible spectral range, whereas the CD value is negligible compared to ORD. The black dashed line represents zero CD and ORD values.

The Kramers–Kronig relations (eq 2) are illustrated in Figure 1 by model spectra of a typical proteinic α helix, with its CD absorption bands in the UV and dispersion-like bands for its ORD. Note that CD is completely negligible in the visible, whereas ORD remains significant but varies very slowly in this spectral range. Equation 2 implies that the measurement of a complete ORD spectrum would provide all the information needed to deduce the CD spectrum, and vice versa through the conjugated relation. An immediate consequence of eq 2 is that an ORD measurement in a restricted spectral region, particularly one that does not contain all CD bands, cannot provide the information required to deduce the full CD spectrum. Although an ORD spectrum is relatively easy to measure in a transparency region, it thus lacks the chemical information encoded in the CD bands in the absorption regions. The CD spectrum of protein molecules is related to their secondary structure, including α helices and β sheets,^8−10^ and provides much detailed information about these biomolecules. This information cannot be recovered from a limited wavelength range of the ORD spectrum, however strongly it may be enhanced by plasmonic interactions.

We now move from molecules to much larger nanoparticles. Plasmonic nanoparticles, in particular, interact very strongly with light, thanks to their high density of free electrons and their resonances at wavelengths specific for each particular nanoparticle’s material, size, and shape. It is tempting to try and enhance the weak optical chirality effects of molecules by coupling them to plasmonic nanostructures,^11^ chiral or otherwise. Plasmonic nanostructures^12,13^ can be nanofabricated in chiral shapes or synthesized in the presence of chiral molecules or under conditions breaking mirror symmetry. Such chiral nanostructures are expected to interact differently with molecules of different chirality, which may open opportunities to detect or to separate enantiomers. Chiral plasmonic nanostructures themselves will interact differently with left- and right-circularly polarized light through their localized surface plasmon resonances, which will give rise to much larger optical chirality signatures than those of molecules,^11^ and make single-nanoparticle analysis of the chirality possible. Alternatively, weak chiral optical effects of molecules might be amplified by the strong local fields produced in resonant plasmonic structures, which themselves might not necessarily have to be chiral. Additionally, the strong confinement of optical fields in plasmonic nanostructures is an attractive feature that might enable chirality analysis with much smaller quantities of a substance than are currently required by CD spectrometers, perhaps even down to the single-molecule level. The idea to exploit plasmonic resonances to enhance optical signatures of chirality has inspired a number of review and perspective articles,^5,11,13−17^ which approach the subject from different points of view. However, the combination of a high spatial resolution with a high control of polarization is fraught with experimental and theoretical difficulties, which we aim to explore in this Perspective.

In the above, we have discussed direct scattering, extinction, or absorption measurements of CD and ORD, which are matrix elements of the polarizability tensor. Another less direct way to characterize chirality is to look at the ellipticity of the fluorescence of chiral molecules^18−20^ or of the photoluminescence of chiral nanoparticles.^21,22^ Although these measurements are easier to perform on a single-molecule basis, we will not discuss them in this Perspective, because they have different experimental requirements than scattering and because their relation to the polarizability tensor is complex. Indeed, the chirality of a molecule when it absorbs may differ from its chirality in the emitting relaxed state.

## Experimental Hurdles

The optical analysis of the chirality of a single plasmonic nanostructure differs considerably from the extinction measurements on large ensembles of identical but randomly oriented molecules in solution, which are routinely done with commercial CD spectrometers. This problem requires considerable experimental advances with respect to current experimental techniques. In a conventional CD spectrometer, a weakly focused, polarized light beam propagates through a solution of the chiral analyte, and the polarization state of the transmitted beam is carefully measured. Recovery of the analyte’s CD spectrum from the beam’s polarization state relies on the following hypotheses:

(i) Analytes are identical and so small that scattering is negligible, and the beam’s extinction stems exclusively from absorption. This hypothesis is clearly unsuited for nanoparticles, which are all different in size and shape and which often scatter light if they are not much smaller than the wavelength.
(ii) The light beam of a standard spectrometer has a small numerical aperture (NA) and can be easily separated from any secondary emission from the sample, in particular, from light scattered by the analytes. This assumption is hard to fulfill in the case of an optical microscope, particularly when CD analysis should be combined with images at high spatial resolution of single nanostructures, and therefore, illumination and collection must be performed with high numerical apertures and large off-axis angles. Moreover, the beam polarization at the focus of a microscope is notoriously difficult to control precisely and is very sensitive to small misalignments and imperfections.
(iii) ORD effects (polarization rotations) can be ignored in standard CD spectrometry because they are rejected from CD signals by a dual modulation technique. In the case of a single nanostructure, the non-negligible scattered waves also carry an ORD contribution as well as a CD contribution, and these ORD effects should be included in the analysis of the signal. A similar effect arises in the Mie theory of scattering by a metal sphere, where the extinction cross-section involves both parts of the complex dielectric permittivity, whereas the absorption cross section only involves its imaginary part.
(iv) The absorbing entities are oriented randomly and isotropically in space. However, a single nanostructure or even an ensemble of nanostructures are often grown, fabricated, or deposited on plane substrates and are almost never randomly oriented. Unless they are averaged in six spatial directions,^5^ measurements on single nanostructures will be orientation-dependent. Moreover, nanostructures usually present a strong linear dichroism (LD), which would disappear in the orientational averaging of the solution, but can easily dominate weak CD effects in single-structure measurements.

**Figure 2:**
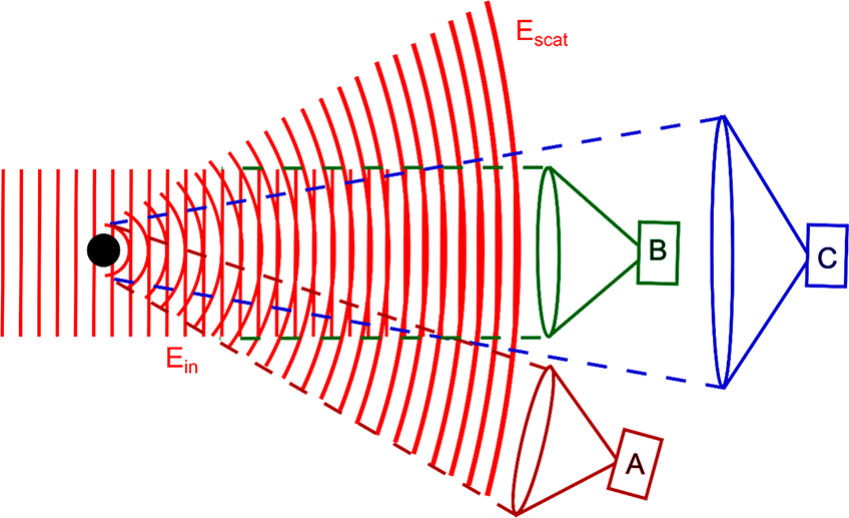
Schematic representation of three detection configurations of scattering signals that may convey CD information: (A) dark-field scattering, (B) bright-field scattering corresponding to extinction, as used in conventional CD spectrometers, and (C) hybrid detection configuration that may apply to a high-NA collection in an optical microscope. The latter signal consists of components from bright-field and dark-field configurations. *E*_in_ and *E*_scat_ are incident and scattered fields, respectively.

Because these four requirements cannot be met for measurements on single nanostructures and even often for large numbers thereof, the relation between the true CD spectrum and the signals measured in an extinction configuration is highly nontrivial. Whether the measurements are based on a standard CD spectrometer or are obtained by means of a high-NA optical microscope, they have to be evaluated and interpreted carefully in each case. Directly using the CD spectrometer output as CD spectrum is only granted if the nano-objects are small (typically below 30 nm in diameter) and if they are randomly oriented in space.^23^ Moreover, if the same polarization techniques are applied in an optical microscope with high numerical aperture, the concomitant effects of extinction and scattering must be considered^24,25^ (see Figure 2) and the role of ORD in scattering cannot be ignored. For large nanostructures, a direct measurement of absorption^26−29^ free from any scattering contributions is desirable. Photothermal circular dichroism (PT CD) microscopy^30,31^ enables such CD measurements purely based on absorption, as we will discuss later in this Perspective (see Measurements of Single NPs).

## Single Plasmonic Nanoparticles (NPs)

Measuring the circular dichroism spectrum of a single molecule appears well beyond our current experimental abilities. Therefore, a first, easier step is the measurement of the CD spectrum of single chiral plasmonic nanoparticles. In the following, we consider only metallic plasmonic particles, although dielectric particles and in particular their magnetic resonances^32,33^ could present interesting chirality properties. Chiral plasmonic nanoparticles are typically as large as or larger than biomolecules, and thanks to their plasmon resonances, they can interact very strongly with light. Chiral nanoparticles can be nanofabricated using electron-beam lithography,^34,35^ chemically synthesized,^36,37^ or assembled through interaction with peptides^38,39^ or with DNA templates.^40−42^ Several optical techniques can be utilized to measure CD and possibly ORD signals of single plasmonic particles, as has been experimentally demonstrated in the past few years.^26,31,43,44^ Instead of reviewing the pros and cons of these techniques, we hereafter list particular points that apply to all of them.

(i) Individual nanoparticles differ by size and shape, and their plasmonic properties strongly depend on these two parameters. Therefore, it will be difficult to obtain a general understanding from measurements of only a few individual nanoparticles. Extensive measurements of many individual nanoparticles are required to gain insight into the distribution of their CD properties and its dependence on synthesis conditions.
(ii) At the level of individual particles, one has to consider the orientation of the nanoparticle with respect to the axes of the optical measurement system. Such experimental parameters are typically averaged out in measurements on solutions or suspensions. A full characterization of a nanoparticle’s chiral properties requires sampling of its orientations or at least an average over three (*x*, *y*, *z*) or better six (±*x*, ±*y*, ±*z*) orientations^5^ to reproduce the ensemble-averaged result.
(iii) The intensity of light scattered away from the incident light beam is negligible for small molecules, but the (dark-field) scattering cross-section of a plasmonic nanoparticle scales quadratically with its volume. Therefore, nanoparticles with sizes larger than typically 30 nm in diameter scatter a significant amount of light, so that extinction can no longer be assimilated to pure absorption. If extinction is measured, a proper theory should relate the measured extinction and its dependence on the circular polarization state to the true CD arising from pure absorption. Figure 2 shows different cases of possible signal integrations at the output of an optical microscope that can provide the extinction (Figure 2A), the dark-field scattering signal (Figure 2B), or a superposition and possible interference of both these signals if the detector covers a larger solid angle than the transmitted beam alone (Figure 2C). More complex collection types have been developed for scattering (iSCAT) microscopy,^45^ and each particular one of them ought to be considered for quantitative evaluation of the data, if they are used to enhance polarization-dependent signals.
(iv) A considerable difficulty in CD measurements of single particles is the possible leakage of their (often) strong linear dichroism (LD) into the CD signal.^46^ The LD signal is averaged out in measurements on large ensembles in solution by the isotropic distribution of particles. However, this cancellation does not take place for a single particle. The LD signal, which can contaminate the CD measurement through imperfections of the optical detection system or by slight misalignments, should be carefully monitored and eliminated. This elimination of LD is usually performed by a double modulation of the polarization state, which removes LD by combining measurements done in two orthogonal systems of axes.^47,30^
(v) Most chiral plasmonic nanoparticles have CD bands in the visible that obviously are associated with ORD bands in the same spectral range. Whenever a scattered field contributes to the measured signal, it may be important to consider ORD rotations of the field polarization in addition to the CD ellipticity, in order to evaluate the optical signal quantitatively. This problem does not arise when a pure absorptive signal is obtained, as done in photothermal microscopy.
(vi) A final point concerns the complex relation between the structure of a nanoparticle at different scales and its optical activity.^48,49^ Whenever the overall shape of a chiral nanoparticle differs from its mirror image, one has a geometrical chirality known as Hausdorff chirality.^50^ Structural or chemical defects in the bulk or at the surface of the particle may also lead to a chiral response. Such effects from weak structural deviations, which would be averaged out in measurements of large ensembles of nanoparticles, may not be neglected for single nanoparticles. To our knowledge, no clear relation has been found so far between the chiral signal of a particle and its overall shape, surface defect, and protrusions.^51^

Because of the strong collective coupling of electrons to light in plasmonic particles, their chiral response is much stronger and easier to detect than that of single (bio)molecules. Therefore, single plasmonic nanoparticles can serve as convenient specimens to explore new experimental methods to measure and analyze CD and ORD responses. However, the specific properties of NPs and the need for high numerical apertures bring about several complications in the experimental methods needed that should not be overlooked when studying the more complex systems discussed in the following two sections.

## Enantiomer-Driven Assembly of Plasmonic NPs

Nanostructures made out of plasmonic NPs are called plasmonic assemblies and have the potential advantage to concentrate the field down to very small hot spots in small gaps between metallic particles. Analyzing molecules placed in such hot spots, ideally with full control of their position and orientation, would significantly reduce the amount of molecules needed for a CD or ORD analysis. Two fundamentally different paths have been proposed to detect the chiral response of molecules with plasmonic assemblies.^11^ The first route is to let enantiomers of one kind of a chiral molecule drive the assembly process, which is expected to confer a chiral character to the assembly. Although this chiral character, being partly random in nature, may accidentally induce right- or left-handed chirality at the single-assembly level, an ensemble-averaged chirality should emerge upon averaging over a large number of assemblies and would correlate with the chirality of the molecular enantiomer used for the assembly. The second route is the analysis of the chirality of prefabricated assemblies or structures in the presence of molecular enantiomers and will be discussed in the next section.

Let us thus imagine that chiral molecules themselves have driven the assembly of plasmonic NPs. The strong plasmonic interactions between NPs in the assembly lead to strong chiral optical signals. Chemical interactions between chiral molecules and plasmonic nanoparticles can create nanoassemblies whose chirality is specific of that of the molecular enantiomers used, resulting in an enantioselective CD spectrum.^52−56^ Plasmonic nanoparticles such as gold NPs can assemble into helical structures in the presence of chiral molecules. The helix handedness depends on that of the chiral molecules. Let us stress here that the CD signal of the chiral assembly is mostly due to the chiral structure of the assembly rather than due to the much weaker intrinsic chiral response of the scaffold molecules. Such chiral nanoassemblies have the advantages that, first, a signature from chiral molecules can be measured at visible wavelengths and, second, a reduced amount of chiral molecules is often sufficient to detect even weak enantiomeric enrichments, as the molecular chirality is transduced and amplified into a strong plasmonic CD signal. However, detecting chiral molecules with such assemblies requires prior knowledge of the chiral molecules used. Assemblies cannot be used to investigate the conformations and the optical response of unknown samples. Moreover, this detection scheme only applies to ensemble measurements over very large numbers of nanoassemblies. Indeed, single assemblies are too sensitive to the actual defects of the particles, to the details of the gaps, and of molecule–NP interactions for quantitative exploitation at the single- or few-molecule levels. The source of the CD signal is hard to assign to the constituent particles, to their coupling, or to a possible contribution of the molecules themselves. In other words, an enantiomer-induced NP assembly acts as an amplifier for the molecular chirality, but the relation between the two chirality sources is complex and difficult to unravel.

## Molecules at Prefabricated Plasmonic Nanostructures

In this section, we discuss plasmonic structures onto which chiral molecules have adsorbed or have been conjugated by specific linkers. For example, the plasmonic structures can be fabricated on a rigid substrate in a top-down process or be released in solution after such a fabrication step. In all cases, we exclude any backaction of these molecules on the supposedly perfectly rigid metal scaffold. Because of electromagnetic enhancements in hotspots of the plasmonic structure, such constructs have the potential to reduce the number of molecules needed for a CD analysis dramatically compared to a conventional spectrometer.^15,57^ Hereafter, we examine different versions of this idea.

### UV CD Enhancement by Plasmonic Nanostructures

The most straightforward scheme to downsize the interaction region between the chiral molecules and the field is to concentrate the electromagnetic field^58^ used in conventional CD spectrometry of biomolecules thanks to plasmonic antennas. Because biomolecular CD is measured in the electronic absorption range of small molecules, in particular, amino acids and nucleotides, this scheme must concentrate waves in the UV spectral range (200–350 nm) for which the standard plasmonic metals, gold and silver, are strongly absorbing. Aluminum appears to be the best candidate^59^ as a plasmonic material in the UV range, although its use raises many issues of nanofabrication, stability with respect to oxidation in ambient conditions, and of the difficult synthesis of Al nanoparticles.

An even more serious problem is the irreversible photochemistry that biomolecules would undergo under intense UV irradiation. This photodamage is not an issue for ensemble experiments on the very large number of molecules in standard CD spectrometry, but it will become a fundamental limitation for single-molecule CD in the UV. Further experimental difficulties would arise in controlling polarization purity and spatial resolution in the UV, for which sources, optics, and detectors are not as developed as in the visible range. A further complication, mentioned above, would be the interplay of ORD and CD plasmonic spectral responses with the CD response of the molecules. Complex optical near-field simulations would be needed to understand and exploit the full optical response of the complex metal–molecule composite structure.^41,60^

Assuming the above problems solved, we may speculate on the optimal design of a metal nanostructure for UV plasmonic enhancement. Ideally, the structure would have to enhance both the electric transition dipole and the magnetic transition dipole, while keeping them aligned, or at least nonorthogonal (see eq 1). Standard plasmonic cavities do not appear well suited to that goal. A dimer of metal particles, for example, enhances interactions with an electric dipole directed along the gap, but does not present the right geometry to enhance a magnetic dipole. Similarly, a ring structure or a dielectric nanosphere may enhance optical magnetic fields that interact with a magnetic dipole, but cannot be easily combined with enhancement of an electric field perpendicular to the ring that would interact with a linear electric dipole. Dedicated structures would have to be studied and fabricated that enable both enhancements simultaneously.

### Chiral Response Enhanced by Superchiral Fields

A related approach is to try and develop near-field optical configurations that enhance not only the electric and magnetic fields, but a proper combination of them that would enhance interactions with chiral objects, so-called superchiral fields. Those fields could be created by the illumination of plasmonic structures^61−64^ or of dielectric nanostructures.^65^ Assuming these superchiral fields have been created, one would have to position the molecule in the active region and control its interactions with the structures and, in particular, the molecular orientation. Indeed, a chiral hot spot would interact differently with the two enantiomers of a molecule. And this would make it hard to distinguish which enhancement comes from chemical interactions and which comes from electromagnetic interactions. A similar problem has existed for many decades in the field of SERS (Surface Enhanced Raman Scattering)^66^ and is still not satisfactorily solved. If the field in the plasmonic hot spot itself is not chiral, its response could become chiral when chiral molecules are placed in the hot spot. Therefore, a more conservative strategy, discussed in the next subsection, is to start from an achiral hot spot and to investigate the chiral signals that report on the handedness of molecules placed in the hot spot. Alternatively, a racemic mixture of chiral structures,^67^ which would enhance signals of both enantiomers, would enable the detection of weak enantiomeric enrichments.

### Plasmon-Coupled CD (PCCD)

Here, we consider NPs, NP assemblies, or nanostructures that are achiral and, therefore, do not, in first approximation, produce any CD or ORD signal. Only when a chiral molecule, or a small number of them, is placed in suitable hotspots do these structures yield a measurable CD or ORD signal. The near field of the achiral structure is coupled to the chiral molecules, which imprints a chiral signature to the total response. The coupled response is expected to be much stronger than that of the isolated molecule. A typical case would be a CD response of a composite formed by a plasmonic system absorbing in the visible spectral range and by a biomolecule with a pure ORD response in the visible. We can interpret this effect in a naïve way as arising from the sensitivity of the plasmonic response to the index of refraction of its surroundings. The plasmon absorption spectrum of the composite will shift under a left-circularly polarized (LCP) illumination. This shift will differ from the shift under a right-circularly polarized (RCP) illumination, because the molecules respond with different refractive indices to LCP and RCP. Subtraction of the differently shifted plasmon absorption spectra will produce a derivative-like or dispersion-like absorption profile for the CD response of the composite. Although this naïve interpretation cannot replace a full electromagnetic simulation, it qualitatively shows the physical origin of measured signals.^68−71^

**Figure 3:**
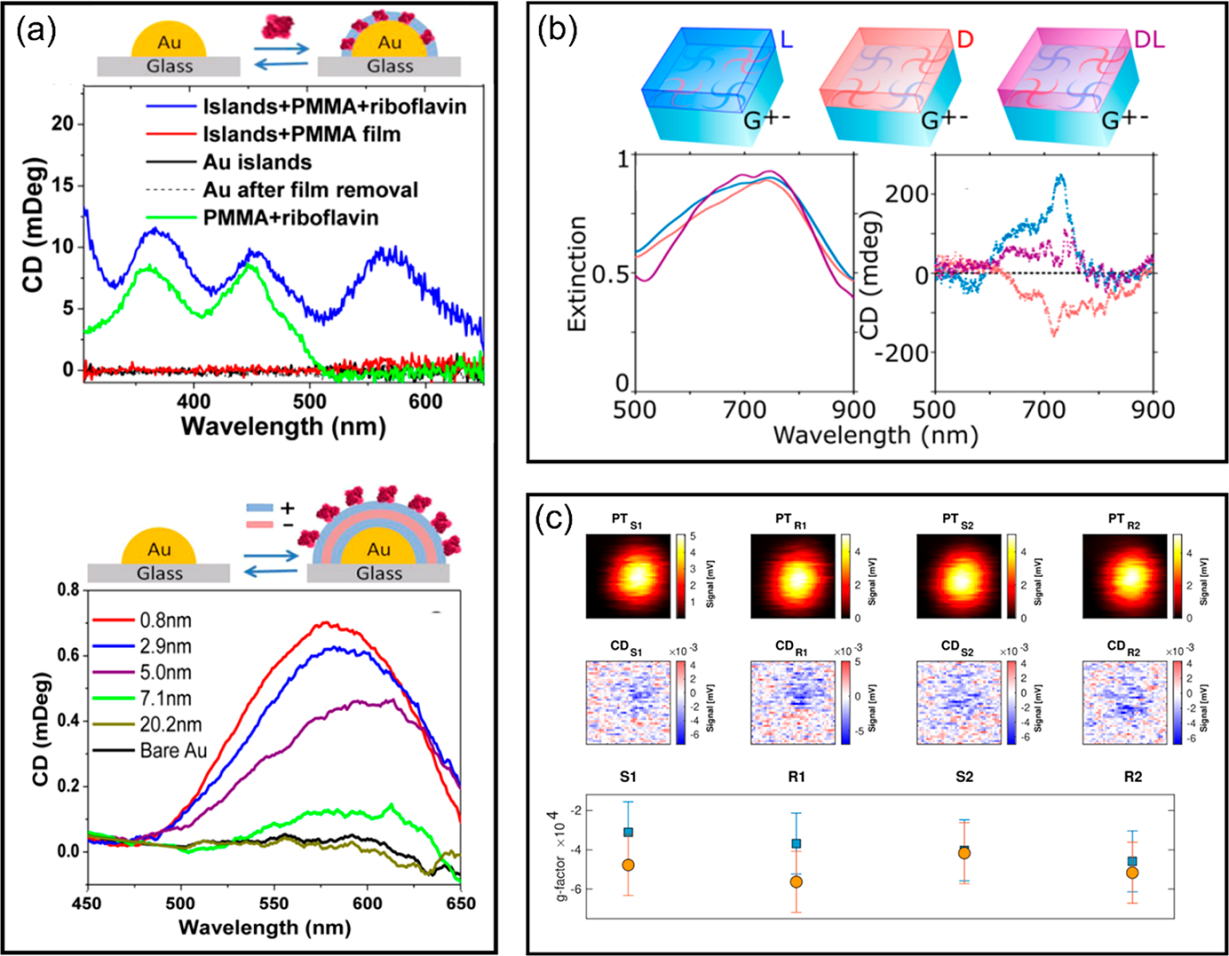
(a) Top: CD spectra of riboflavin in the absence and presence of gold nanoislands showing the appearance of a plasmon-coupled CD (PCCD) signal in the wavelength range of the plasmonic absorption of the gold islands. Bottom: the PCCD signal increases when decreasing the thickness of the spacer layer between the gold metal and the riboflavin molecules, showing that the PCCD is a near-field effect. Adapted with permission from ref (70). Copyright 2013 American Chemical Society. (b) Extinction and CD spectra of phenyl-alanine enantiomers: d-enantiomer, l-enantiomer, and dl racemic mixture in the presence of an array of left- and right-handed gammadion structures. Strong CD signals appear in the near-IR range for either enantiomer, but nearly cancel for the racemic mixture. Reproduced with permission from ref (72). Copyright 2018 American Chemical Society. (c) Photothermal (PT) and CD images of a single gold nanoparticle immersed in the *R*- and *S*-enantiomer of the chiral solvent carvone. No significant change in CD signal is observed from one to the other enantiomer, indicating that the PCCD effect has a *g*-factor lower than 10^–4^. Reproduced with permission from ref (30). Copyright 2021 American Chemical Society.

Ideal PCCD experiments ought to compare the signals from left- and right-handed enantiomers of the same compound. Note that this requirement is impossible to fulfill with large biomolecules, because only one (homochiral) enantiomer is available. Even for small molecules, few reports to-date comply with the above requirement. Maoz et al.^70^ found a clear PCCD effect by measuring CD signals in the visible spectral range for gold nanoislands in the presence of chiral riboflavin molecules, whereas the same gold islands, bare, had no measurable CD in the visible (Figure 3a). The measured CD varied according to the position of riboflavin in a layer-by-layer deposit on the gold island surface, neatly demonstrating the near-field origin of this PCCD effect. Garcia-Guiardo et al.^72^ demonstrated a PCCD effect in a series of experiments on a racemic array of gammadion nanostructures (Figure 3b). In the presence of either d- or l-enantiomers of phenylalanine, the array showed enantioselective CD spectra, which vanished in the presence of the racemic molecular mixture. PCCD experiments have been attempted on single particles in the authors’ group^30^ (Figure 3c). Single gold nanospheres were immersed in the chiral solvent carvone, but no PCCD effect could be detected at the level of a few 10^–4^, below the detection sensitivity. The PCCD effect can be estimated to about 10^–4^ from chiral Mie theory.^73^ This low value is due to the comparatively weak ORD of carvone.^74^ The experiment should be repeated with a liquid with a higher ORD, such as binaphthol.

Concluding the sections Enantiomer-Driven Assembly of Plasmonic NPs and Molecules at Prefabricated Plasmonic Nanostructure, assemblies of plasmonic nanoparticles offer attractive enhancements of the chirality and might enable detection of small amounts of chiral molecules or of weak imbalances in nearly racemic mixtures. However, these advantages only appear at the ensemble level, because the many defects and complexities of these assemblies cancel in the averaging of a large number of similar systems. Therefore, assemblies of nanoparticles appear to us of little use for the study of individual assemblies and, ultimately, individual molecules, because they are extremely sensitive to imperfections and defects.

## Measurements of Single NPs

### Ensemble Experiments

Most measurements on chiral objects so far were done on large ensembles with many objects. Beyond the sheer addition of the many very small signals from each individual object, a key advantage of ensemble averaging is that it eliminates many artifacts. As stated above, effects such as orientational averaging and strong LD contributions to the weak CD signal should be considered carefully in single-particle measurements.^46^ Similarly, random shape irregularities of nanoparticles are expected to cancel on average in ensemble measurements, so that the signature of the average shape, in particular a chiral shape, can emerge from the averaging. However, many interesting details of the nanoparticle-molecule interaction would also be averaged out and therefore would disappear from such ensemble experiments.

### Single-Particle Experiments

To retain details of the chiral properties of nanostructures and of their interactions with chiral molecules, it is essential to suppress the averaging of ensemble measurements and to attempt single-particle measurements.^30,31,34,43,44,68,75−77^ All single-particle measurements performed so far have found a pronounced heterogeneity in the CD signals of individual particles. Single-particle photothermal CD spectroscopy of single nominally spherical gold nanoparticles found that CD signals vary from particle to particle.^30,31^ Similar heterogeneous behavior has been observed for single plasmonic oligomers,^34^ gold helicoids,^75^ and single gold nanorod dimers or assemblies^43,68^ using single-particle dark-field scattering microscopy. Single-particle extinction spectroscopy enabled measurement of CD spectra of single aggregates of mercury sulfide (HgS) nanocrystals.^44^

**Figure 4:**
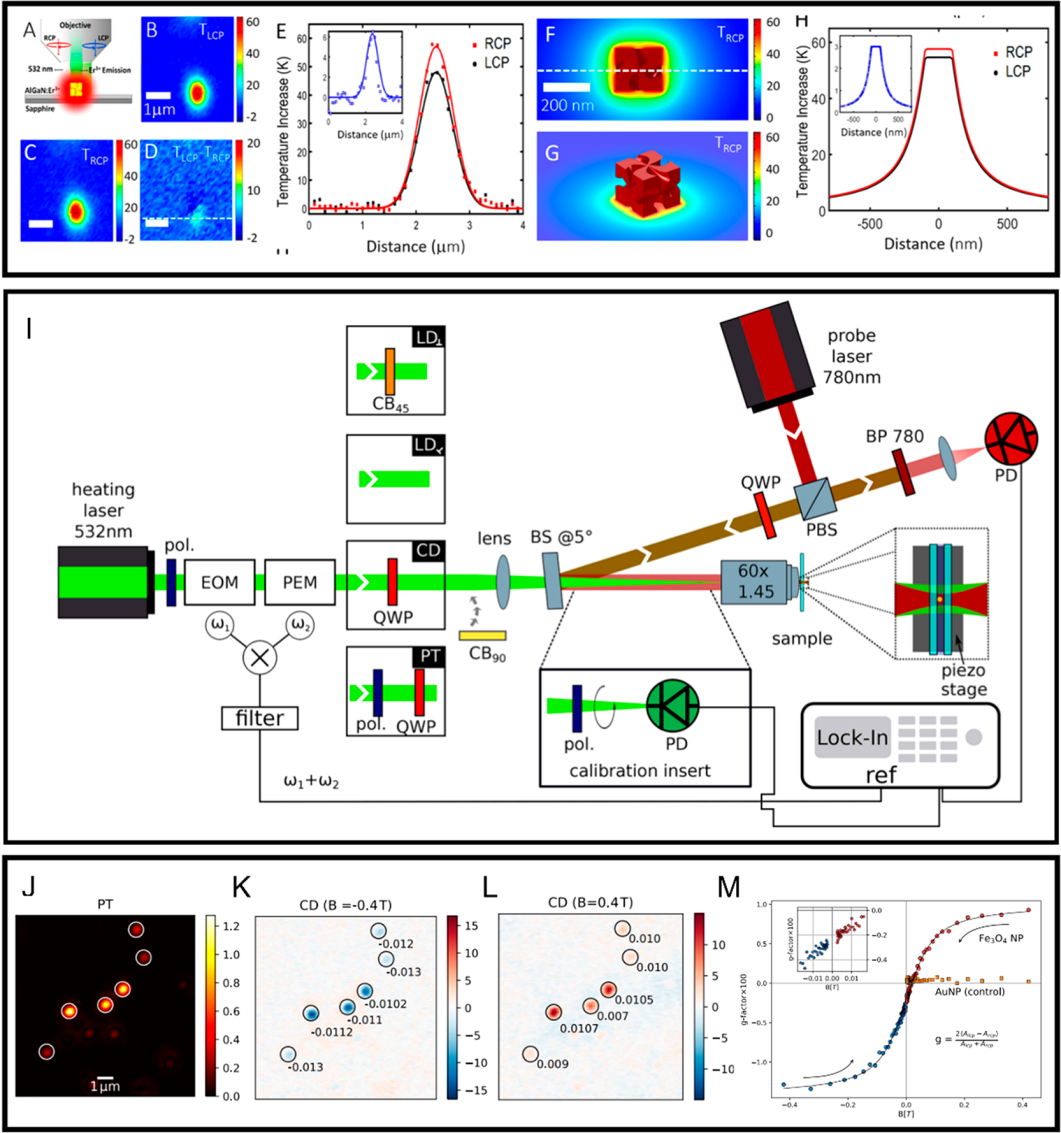
(Top panel, A–H): (A) Scheme for photothermal nanothermometry. The temperature map of a helicoid nanocluster with left- and right-circularly polarized light is shown in B and C, respectively. The temperature difference map reporting on the CD is shown in D. The corresponding temperature profiles are shown in E. The inset in E shows the line profile along the white dashed line in D. (F–H) Thermal image and profiles of a single helicoid using COMSOL simulation. Adapted with permission from ref (26). Copyright 2020 American Chemical Society. (Middle panel, I) Scheme of a photothermal circular dichroism microscope. Reproduced with permission from ref (30). Copyright 2021 American Chemical Society. (Bottom panel, J–M): (J–L) Photothermal (PT) image and CD images at magnetic fields of −0.4 and 0.4 T of single nanoclusters of magnetite nanoparticles, respectively. (M) The magnetization curve of a single nanocluster shows superparamagnetic behavior, whereas a gold nanoparticle shows diamagnetic behavior. Adapted with permission from ref (84). Copyright 2022 American Chemical Society.

Many authors follow the classical extinction configuration of conventional molecular CD spectrometers, that is, they collect light transmitted through a sample containing the particles to analyze. Applying this configuration to optical microscopy with large numerical apertures to improve spatial resolution and study smaller objects leads to the difficulties discussed in the sections Experimental Hurdles and Single Plasmonic Nanoparticles (NPs) (see Figure 2). Extinction is a particular case of the bright-field scattering configuration, where the detected beam is matched exactly to the transmitted beam, sometimes to the beam reflected by a nearby interface. Whereas CD is defined as the difference in the absorption of left- and right-circularly polarized light, differential scattering is much more complex, because it involves interference terms between the incident field and the scattered field. The phase difference between these fields itself becomes relevant because of Gouy phase shifts and mode-matching factors and, in principle, involves details of the optical configuration that are of no concern in the conventional configuration of solution CD spectrometry. Thus, a full treatment of differential extinction depends on the ORD properties in addition to the CD of the particles. Therefore, it is not clear how a differential scattering spectrum relates to the “true” CD spectrum of the same object measured with an ideal CD spectrometer with low numerical aperture (NA) and infinite signal-to-noise ratio. Moreover, measurements with high NA are prone to polarization imperfections because of the complex polarization structure of a tightly focused light beam.^78^ Therefore, a direct measurement of differential absorption is highly desirable, as it obviates the problems of the collection of scattered light and of contamination of the signal by ORD. Such a technique exists for very small particles, this is photothermal CD detection (PT CD), which we discuss hereafter (see Figure 4I). This method, first proposed by Kitamori^27^ for microfluidics, has been more recently adapted to microscopy of immobilized particles by ourselves^30,31^ and by Miandashti et al.,^26^ who applied it to photothermal CD measurements of single gold helicoids using luminescence thermometry (see Figure 4A–H). Theoretical work on photothermal CD was published by Kong et al.^28^

### Photothermal Circular Dichroism Microscopy

The most general measurement of the chiro-optical response of a nanoparticle would provide both ORD and CD, related respectively to the real and imaginary part of the dielectric permittivity tensor. As we have seen above and in Experimental Hurdles, doing such a measurement in an optical microscope brings about a number of complications. However, if we can measure a consequence of absorption, such as the temperature change caused by optical absorption, we can separate the CD signal induced by the imaginary part of the permittivity tensor, free from any ORD contribution. PT CD microscopy is based on a nonlinear optical pump–probe technique called photothermal microscopy.^79^ The pump (or heating) beam’s polarization is modulated at a high frequency, typically some tens or hundreds of kHz, between left- and right-circularly polarized light. A chiral nanoobject will absorb more light of one handedness than of the other, depending on the object’s handedness and on the light wavelength. Heating light absorption by the chiral object is followed by nonradiative relaxation, and the heat released creates a temperature gradient in the surrounding medium. The temperature of the object itself also changes, but for a small enough object, the change in its optical properties is often negligible against that of the surrounding medium, whose index of refraction is modified by the temperature gradient. This region of changed index, called the thermal lens, scatters a second beam, the probe beam, whose wavelength is often chosen out of the absorption region of the object to be detected. The scattered probe light interferes with the reflected or transmitted probe beam that acts as a reference, much as is done in interferometric scattering, iSCAT.^45,80^ As the interference signal varies at the pump beam’s modulation frequency, it is picked up sensitively by a lock-in amplifier. To isolate the CD signal, one must remove polarization artifacts very carefully.^81−83^ A key advantage of PT CD compared to other chirality-sensitive single-particle microscopy methods based on scattering or extinction is that PT CD only cares about the polarization of the heating light, not at all about that of the probe light. Therefore, the probe can be tightly focused with a high NA, so as to preserve a high spatial resolution. The heating beam in a Koehler illumination configuration is much more loosely focused, which enables a strict control of its polarization quality. PT CD microscopy makes use of two polarization modulators, a scheme also used in commercial CD spectrometry (Figure 4I). These modulators set at frequencies ω_1_ and ω_2_ produce a PT CD signal at ω_1_ ± ω_2_. Isolating one of these frequencies, the lock-in amplifier rejects polarization and LD artifacts that would contaminate the signal under modulation at a single frequency. With dual polarization modulation, we could detect CD signals of single particles with a sensitivity of 3 × 10^–4^ in *g*-factor (ratio of CD to unpolarized absorption). Nominally spherical (thus, nominally achiral) gold nanoparticles show weak CD signals at the single-particle level, which can be measured with high sensitivity thanks to the PT CD technique. The source of this CD signal is not well understood. It could stem from non-mirror-symmetrical deviations from a spherical shape, surface roughness, or even lattice defects. Much remains still to be done in single-particle PT CD microscopy to understand the origin of chiral signals. Note hereby that, for the large plasmonic nanostructures, which would be required for strong PCCD enhancement, the probe scattering by the nanostructure will interfere with the scattering by the thermal lens. Therefore, in the absence of a complete theory, a compromise will have to be found between a strong plasmonic enhancement and a moderate contribution to the scattered intensity.

A particularly interesting and important application of chirality studies is magnetic-field-induced chirality (see Figure 4J–M). As is well-known, magnetic systems are not mirror-symmetric unless a time-inversion is performed along with mirror reflection. Application of a magnetic field therefore induces a *controllable* chirality that can be detected via the polar magneto-optical Kerr effect (MOKE) with a PT CD microscope. Our group recently published studies of superparamagnetic magnetite nanoparticles,^84^ but many other magnetic nanostructures could be studied in the same way.

## Conclusion and Perspectives

The dream of measuring the CD spectrum of a single biomolecule and thereby to access its conformation remains very remote. The UV regime is a difficult one for light sources, optics, and detectors. Moreover, photochemistry caused by necessarily heavy UV illumination of small numbers of molecules will severely limit the acquisition of weak optical signals. This issue of photostability does not arise for the large ensembles of molecules probed with conventional CD spectrometers. Today’s limits of sensitivity still fall by several orders of magnitude short of single-molecule sensitivity in CD spectrometry because of the weakness of the CD signals. In our discussion of CD and ORD responses of single small objects, we encountered a number of important additional points. Dealing with optical signals from single nano-objects hits specific experimental hurdles, which do not arise for measurements on large ensembles in solution:

(i) Optical microscopy of small objects requires high numerical apertures (NA), which are not readily compatible with a high polarization purity.
(ii) High NAs make it hard to distinguish dark-field scattering from bright-field scattering and from extinction; the exact admixture of these signals will depend on the experimental configuration used (see Figure 2). That means that a quantitative exploitation of chirality measurements in extinction configurations may turn out to be impractical.
(iii) On single objects and even on small numbers of them, linear dichroism (LD) does not cancel through orientational averaging, so that LD artifacts may easily dominate weak CD signals.
(iv) Finally, depending on the scattering and extinction amounts in the signal, ORD may also enter the signal in addition to CD.

Plasmonic nanostructures have been proposed as possible enhancers for weak chiral signals.^11^ All the points above have to be considered when applying chirality measurements to plasmonic nanostructures. We have discussed the following potential types of nanostructures:

(i) Single plasmonic nanoparticles, nanospheres, nanorods, and so on often are nominally achiral, but present shape, surface, or composition defects that make them chiral. The relation between structure and chiral signals from these particles is still mysterious and its clarification requires further effort.
(ii) Nanoparticles (NPs) can be assembled, notably in view of creating hotspots with high fields that might improve chirality measurements. A first type of NP assembly is those *driven* by chiral molecules, which often do imprint their own chirality onto the obtained assemblies. Plasmonic interactions between particles can thus, in effect, enhance molecular chirality. However, the relation between molecular chirality and the plasmonic response of an assembly is by no means direct.
(iii) A second route to interactions between a plasmonic structure and chiral molecules is to prefabricate an assembly of NPs or a more complex nanostructure. This object can then act as a host for chiral molecules, without any molecule-induced change in its structure. In this approach, an achiral nanostructure delivers a chiral signal, depending on which molecular enantiomer is adsorbed in its hot spot(s).^58^ This scheme could be applied to direct CD measurements in the UV range or to the creation of superchiral fields in hot spots, although unknown specific interactions between chiral structures and chiral molecules may complicate understanding and control of these signals. The most straightforward and conservative approach appears to be plasmon-coupled CD (PCCD), in which an achiral plasmonic hotspot translates the chirality of a molecular enantiomer into an optical signal in the visible range, with a sign associated with each enantiomer. It should be stressed again, however, that PCCD of a biomolecule is by no means a proxy for its CD spectrum in the UV. Indeed, the PCCD signal is rather a signature of the molecule’s visible ORD than a “reflection” or “translation” of its CD spectrum in the UV. As ORD (or, for that matter, CD) is featureless in the visible, it cannot retain the wealth of conformational detail encoded in the UV CD spectrum.

We have discussed an experimental method, PT CD, which solves some of the problems mentioned above, notably the separation of ORD from CD, the separation of absorption from extinction, and the separation of LD from CD. The hardest problems, however, which are the weakness of the signals, their dependence on orientation, and the need for UV microscopy to address relevant biomolecules, remain.

Is it then utterly hopeless to look for single-molecule CD? It certainly is a challenging goal, but we wish to conclude this article with some hopeful ideas. With single-molecule CD measurements, it would be possible to distinguish the two enantiomers of a same molecule through their CD signals at a given wavelength. However, this would require previous knowledge of the sign of the CD signal and knowledge that the molecules are not clustered. Obtaining biomolecule conformational information, however, would still require a full CD spectrum of each molecule. A possible pathway to avoid direct heating and damage of plasmonic nanostructures with resonant UV light might be two-photon excitation of the electronic transitions of biomolecules.^85^ Resonant but nonabsorbing plasmonic nanostructures would be illuminated with visible or near-IR light, with no or very reduced absorption in properly chosen materials. Possible candidate nanoparticles are dielectric nanostructures,^65,86^ whose magnetic resonances could be exploited for magnetic-dipole enhancement. These nanostructures would not be directly heated by UV light resonant with the plasmons. In this scheme, two-photon excitation would directly address the electronic degrees of freedom of the biomolecular analyte, whereas visible or near-IR frequencies would be used to excite the plasmonic dielectric resonances.

Photothermal CD microscopy signals can be boosted by more sensitive photothermal transducing liquids, such as near-critical fluids.^87,88^ The large change with temperature of their specific volume at constant pressure amplifies very small temperature variations, such as those produced by weak absorption differences between right- and left-circularly polarized light. Alternatively, optomechanical devices can measure absorption of small objects with even higher sensitivity.^89^

The problem of photostability of (bio)molecules under strong UV irradiation may be circumvented with vibrational CD,^90−92^ which addresses mid-infrared-active molecular vibrations and bears very detailed structural and chemical-bond information.^93^ Unfortunately, because of the weakness of absorption coefficients in the mid-IR (vibrational CD is about one order of magnitude weaker^94,95^ than electronic CD), the single-molecule regime has not been attained yet in mid-IR vibrational absorption.^96^

As mentioned in the introduction, fluorescence or photoluminescence microscopy can be used to detect chirality.^18−20^ Because of the high sensitivity of fluorescence measurements, the access to single molecules and particles is much facilitated for the restricted class of molecules and nanoparticles that emit significant photoluminescence. However, one again will have to carefully consider polarization imperfections introduced by optical elements, specifically dielectric mirrors. Just as with scattering and extinction-based methods, fluorescence-based methods will require careful control of the polarization of both incident and detected beams.

Whatever the systems studied, researchers will continue looking for new ideas and new schemes to approach the tantalizing goal of single-molecule circular dichroism and optical rotatory dispersion.
